# Development and validation of an open-source pipeline for automatic population of case report forms from electronic health records: a pediatric multi-center prospective study

**DOI:** 10.1016/j.ebiom.2024.105337

**Published:** 2024-09-16

**Authors:** Alba Gutiérrez-Sacristán, Simran Makwana, Audrey Dionne, Simran Mahanta, Karla J. Dyer, Faridis Serrano, Carmen Watrin, Pierre Pages, Sajad Mousavi, Anil Degala, Jessica Lyons, Danielle Pillion, Joany M. Zachariasse, Lara S. Shekerdemian, Dongngan T. Truong, Jane W. Newburger, Paul Avillach

**Affiliations:** a Department of Biomedical Informatics, Harvard Medical School, 10 Shattuck Street, Boston, MA, 02115, USA; b Department of Cardiology, Boston Children's Hospital, Harvard Medical School, 300 Longwood Ave, Boston, MA, 02115, USA; c Division of Cardiology, Department of Pediatrics, Texas Children's Hospital, Baylor College of Medicine, 6651 Main Street Legacy Tower MC E1920, Houston, TX, 77030, USA; d Division of Critical Care Medicine, Department of Pediatrics, Baylor College of Medicine and Texas Children's Hospital, 6621 Main Street, MC E1420, Houston, TX, 77030, USA; e Division of Congenital Heart Surgery, Department of Pediatrics, Texas Children's Hospital, 8718 Linkfair Lane, 77025, Houston, TX, USA; f Computational Health Informatics Program, Boston Children's Hospital, 300 Longwood Avenue, Boston, MA, 02115, USA; g Division of Cardiology, Department of Pediatrics, University of Utah and Primary Children's Hospital, 81 North Mario Capecchi Drive, Salt Lake City, UT, USA

**Keywords:** Data collection, Electronic health records, Multicenter study, Prospective studies

## Abstract

**Background:**

Clinical trials and registry studies are essential for advancing research and developing novel treatments. However, these studies rely on manual entry of thousands of variables for each patient. Repurposing real-world data can significantly simplify the data collection, reduce transcription errors, and make the data entry process more efficient, consistent, and cost-effective.

**Methods:**

We developed an open-source computational pipeline to collect laboratory and medication information from the electronic health record (EHR) data and populate case report forms. The pipeline was developed and validated with data from two independent pediatric hospitals in the US as part of the Long-terM OUtcomes after Multisystem Inflammatory Syndrome In Children (MUSIC) study. Our pipeline allowed the completion of two of the most time-consuming forms. We compared automatically extracted results with manually entered values in one hospital and applied the pipeline to a second hospital, where the output served as the primary data source for case report forms.

**Findings:**

We extracted and populated 51,845 laboratory and 4913 medication values for 159 patients in two hospitals participating in a prospective pediatric study. We evaluated pipeline performance against data for 104 patients manually entered by clinicians in one of the hospitals. The highest concordance was found during patient hospitalization, with 91.59% of the automatically extracted laboratory and medication values corresponding with the manually entered values. In addition to the successfully populated values, we identified an additional 13,396 laboratory and 567 medication values of interest for the study.

**Interpretation:**

The automatic data entry of laboratory and medication values during admission is feasible and has a high concordance with the manually entered data. By implementing this proof of concept, we demonstrate the quality of automatic data extraction and highlight the potential of secondary use of EHR data to advance medical science by improving data entry efficiency and expediting clinical research.

**Funding:**

10.13039/100000002NIH Grant 1OT3HL147154-01, U24HL135691, UG1HL135685.

**Research in context**

**Evidence before this study**

We searched PubMed on April 17, 2024 for publications in the last five years that contained the keywords “data collection” and “electronic health records” in prospective multi-center or observational studies ((Multi-center Study [Publication Type] OR (Observational Study [Publication Type]) AND (((“data collection” [MeSH Terms] AND “electronic health records” [MeSH Terms] AND “prospective studies” [MeSH Terms]) AND (y_5 [Filter]))))). We reviewed the 41 results. Of these papers, 21 were specific to using EHR data to answer specific research questions, six focused on developing machine learning models to predict severity or mortality in different scenarios, and five evaluated quality metrics for clinical care delivery (e.g., time management and time response) using EHR data. The remaining predominantly addressed the development of data analysis tools and the data collection process, including the development of databases used as the source of knowledge for EHRs. Two studies were identified that attempted to use EHRs to fill electronic case report forms. However, these studies did not attempt to apply their technology in ongoing studies, and they focused on validating their results on completed studies.

**Added value of this study**

Repurposing EHR data represents a unique opportunity to speed up data collection and maintain the consistency of the data entry process in clinical trials and registry studies. We have implemented a computational pipeline to automatically populate laboratory and medication forms, two of the most reliable EHR data types, and applied it to an ongoing pediatric multi-centric study. To the best of our knowledge, this is the first study that goes a step further and moves from the validation to actual implementation of an ongoing study. Our pipeline was integrated into an ongoing study as part of the data collection process, running in parallel to the recruitment process, as the research team was focused on collecting other data for the study. Our pipeline also stands out in terms of the potential for broader adoption. The simplicity of the required input file, independent of specific data models, ensures that our methodology can be readily applied across different healthcare settings.

**Implications of all the available evidence**

The impact of our work extends to researchers and patients, addressing a critical gap in current research practices. It primarily benefits researchers and study coordinators, reducing their data entry time and broadening scope. By automating this process, clinicians can focus more on interpreting and analyzing data. Automation also enables studies to encompass broader timeframes and gather additional patient information more easily. Consequently, clinical study participants benefit from improved data quality and consistency, leading to quicker solutions and better health outcomes. Our project tackles a critical and growing issue in clinical research. By automating data entry for some of the most time-consuming case report forms, we propose a transformative solution to enhance research efficiency, quality, and cost-effectiveness. Our open-source approach transcends platform-specific limitations, ensuring broad applicability and generalizability.

## Introduction

Clinical trials and registries can require population by up to thousands of clinically relevant variables, ranging from general demographic information to specific outcomes of interest.^1^ Electronic data capture methods are now widely used, although more than 30% of studies still collect data via paper case report forms (CRFs). Only 20% of companies with electronic data capture software apply electronic health records (EHRs) to clinical trials.^2^ Overall, the data entry process remains primarily manual and commonly requires multiple sources.^3^^,^^4^ In a typical study, researchers gather data, one patient at a time, from various sources, e.g., clinical notes, medical device imaging reports, questionnaires, and EHRs.^5^ Data are manually entered into the study eCRFs. Although standard practice, a manual process has significant drawbacks, including time and resources required for data entry, the sizable estimated error rate of 2.3–26.9%,^6^^,^^7^ and the prevalence of missing information.^8^ There is an urgent need to develop new technologies to simplify processes, decrease the burden of manual data entry, and increase the quality, efficiency, and accuracy of clinical trial and registry data.^9^^,^^10^

When designing new data entry technology, a valuable resource is the EHR. It contains patient-centered demographics, vital signs, diagnostic test results, medications, and clinical notes stored longitudinally as part of routine care. While the EHR was initially designed for billing purposes, recently, the value of leveraging this data for research has become evident.^11^, ^12^, ^13^, ^14^, ^15^, ^16^, ^17^ The EHR has become an essential resource for epidemiological and observational research, especially in retrospective cohort designs. EHR data is used to analyze quality metrics for clinical care delivery, perform clinical research studies, and as a source to power machine learning models.^14^ Although the availability of data in the EHR^18^ and the advantages of its use in prospective studies are apparent,^19^ its use for prospective clinical research has been typically limited to screening for cohort recruitment and clinical feasibility studies.^20^, ^21^, ^22^ Given the overlap between the data collected for research and that collected routinely in the medical records, the EHR data can potentially streamline the data collection. The main challenges are the lack of standardized formats and systems across institutions; the protocol-specific order sets; and data accuracy and completeness.^14^

There is no universal vocabulary or data format that is consistently used across healthcare sites and EHR systems. Because of this, data harmonization and standardization are required to guarantee interoperability and ensure data are uniform in terms of format, structure, and terminology. Several initiatives have emerged to facilitate interoperability. For example, the OHDSI OMOP^23^ initiative aims to create standardized common data models to enable large-scale analysis across healthcare databases. In oncology, the mCODE initiative aimed to standardize some essential cancer-related data elements. Specifically, Fast Healthcare Interoperability Resources (FHIR) serves as a data standard and set of operations, streamlining the standardization and interoperability of EHR data.^24^ Similarly, EHR2EDC^25^^,^^26^ and REDCap^27^^,^^28^ have simulated the performance of automatic and semi-automatic data entry approaches to multi-center collaborative studies. They demonstrate potential for incorporating automation into data entry for research purposes, but lack flexibility and are limited to hospitals with specific EHR models and data capture platforms (e.g., FHIR instances and REDCap). Additionally, the lack of open-source methods in these studies hinders broader adoption.

We aim to develop an open-source pipeline to extract existing EHR data to populate the data requested in the eCRFs, thereby partially automating a large-scale prospective cohort. This article describes the methodology developed. In the context of pediatric populations, we show how we successfully applied the developed pipeline, leveraging the EHRs from 159 participants at two sites, extracting and populating 56,760 laboratory and medication values in an ongoing multi-centric study.

## Methods

### Workflow

**Fig. 1:**
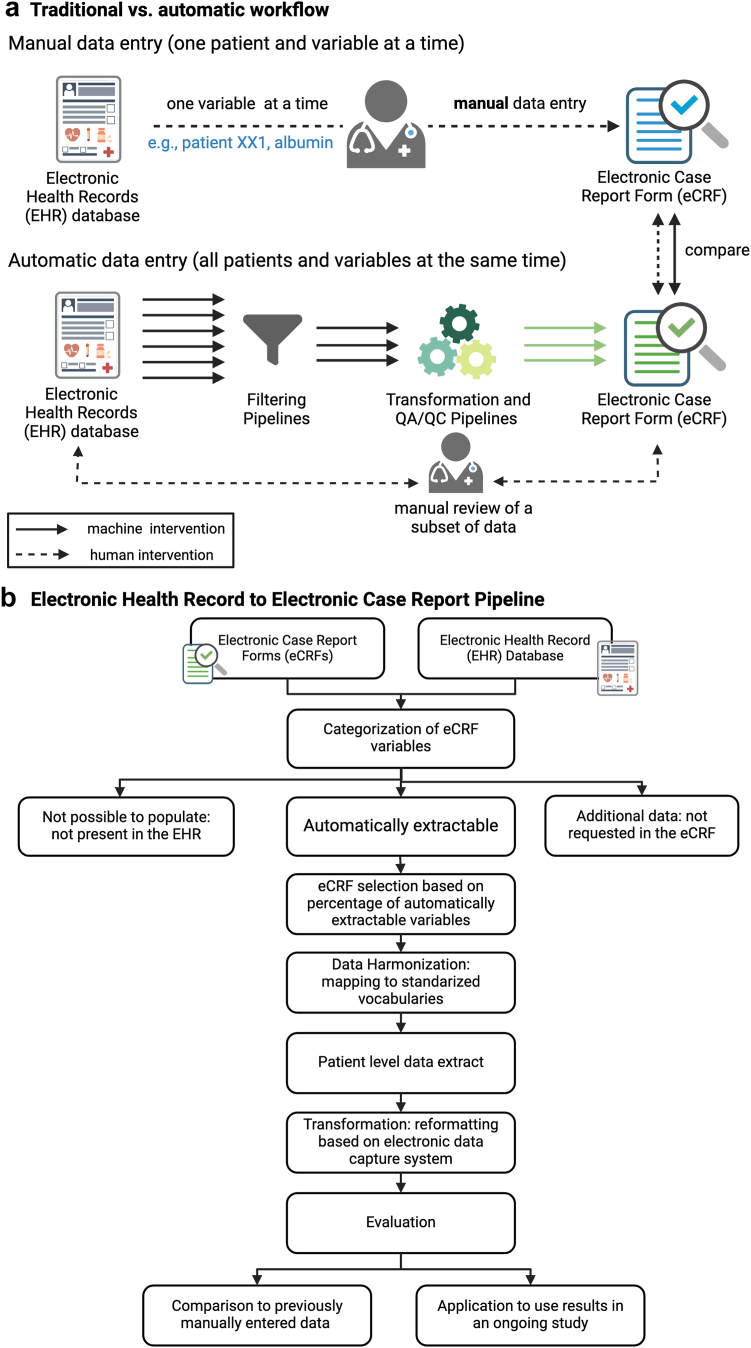
a) Data extraction process: manual versus automatic approach; b) Overview workflow of electronic health record (EHR) to electronic case report form (eCRF) pipeline. Figure created with BioRender.com.

In this prospective study, we developed a pipeline to automatically extract the laboratory and medication data needed for the study using electronic health records (EHR) as a data source (Fig. 1a). The eCRF variables were first categorized by comparing their presence or absence in the EHR. The variables that could be automatically extracted from the EHR were categorized as present in both eCRF and EHR and harmonized to a common language using standardized vocabularies. These steps allowed us to identify the overlap between the variables of interest between the study and the structured data in the EHRs. Then, we developed an extract, transform, and load (ETL) pipeline that allowed us to extract from the EHR patient-level data the variables of interest for the different time points of the study and generate an output file that was ready to load to an electronic data capture system (in this case REDCap) (Fig. 1b).

### Data: COVID MUSIC study

We used the National Heart, Lung, and Blood Institute (NHLBI) prospective study on Long-terM OUtcomes after the Multisystem Inflammatory Syndrome In Children MUSIC study^29^^,^^30^ as a proof of concept to develop our automatic approach to populate eCRFs. COVID MUSIC is a prospective cohort study focused on the long-term outcomes of Multisystem Inflammatory Syndrome in Children associated with COVID-19 (MIS-C). As of January 2022, the study has 1204 enrolled participants in the US and Canada across 31 different hospitals. Two high-enrolling sites (Boston Children's Hospital, BCH, and Texas Children's Hospital, TCH) opted to pilot the automated data collection approach. Due to small patient counts, we randomly renamed the sites A and B for anonymization purposes. In total, 159 MIS-C patients were enrolled for the MUSIC study in the two hospitals. Patient information was collected at admission and discharge for MIS-C, two and six weeks follow-up, and six-month follow-up. Not all the variables were collected at all the time points; some were specific to the admission period.

### EHR data collection and management

The EHR data were extracted from different Clinical Data Warehouses (CDW) at each hospital (i2b2^31^ and Health Catalyst) and stored following a centralized approach. Due to the commonality of the data fields, the required input files can be easily generated independently from the EHR model and the CDW type. Out of all the EHR data, we focused on the inpatient information. Data from each site were organized into two patient-level data files and two dictionary files. The first patient-level file contained patient identifiers and admission and discharge dates. The second included all laboratory and medication observations with timestamps for each patient within the study-defined period. The first dictionary file contained the variables codified as collected at the hospital. The second, when available, contained the map of the variables to standardized vocabularies. Our common vocabulary and file structure are detailed in Supplemental Figure S1.

### Categorization of eCRF variables according to EHR availability/presence

The MUSIC study was designed without consideration for automated data extraction from the EHR. It consisted of 47 eCRFs that were structured text documents containing the names and descriptions of 2449 variables. We first evaluated which eCRFs overlapped the most with the EHR and, therefore, would benefit the most from automated EHR extraction. All eCRF variables were manually reviewed and compared to data found in the EHR. Three case studies were found: 1) variables that were present in both the eCRF and EHR and thus could be automatically extracted, 2) variables that were not present in the EHR and thus not possible to populate, and 3) additional data present in the EHR that was not requested in the eCRF. In this study, we focused on the first category, which is the automatically extractable variables.

### Data harmonization of automatically extractable variables

To determine how many variables of interest were present in the EHR, a crosswalk between the language used at each EHR and the eCRF is needed to identify the variables in common. Data entry varies widely between hospitals, and variables of interest could be described as unstructured text (e.g., free text from medical notes) or structured (e.g., diagnosis, or laboratory test recorded using different coding systems) (Supplemental Figure S2). This study focused on the structured data, specifically laboratory and medication variables. A “common language” or standard vocabulary was required to scale the pipeline to different institutions. We perform a data standardization process to enable comparison between sites and use a format suitable for research purposes. We used the Unified Medical Language System (UMLS)^32^ to map the laboratory and medication variables, collected as structured text in both sites, to LOINC and RXNORM. MetaMap tool and regular expressions based on inclusion and exclusion words were used in the mapping process.

To verify that the structured data elements extracted from the EHR corresponded with the ones defined in the study protocol, we performed a quality control step, summarizing the use of each variable in the EHR. This summary included the first and last time each concept was used, the number of patients and observations with each laboratory test or medication, and the minimum and maximum values and units for the laboratory values (Supplemental Table S1). At this step, we confirmed whether the units used at each institution were the same as required in the eCRF or if a change of units was required. Mappings were verified to be used by each site during the study period.

### Data extraction

In site A, 27,684 laboratory values were extracted from 55 patients. In site B, 24,163 laboratory values were extracted from 104 patients (Supplemental Table S2). The laboratory values were extracted from 75 laboratory tests across five time points. In site A, 1604 medications were extracted from 55 patients. In site B, 3309 medications were extracted from 104 patients (Supplemental Table S2). The medications were extracted from a total of 28 medication categories.

### Data transformation and entry

Once all eCRF-EHR mappings were verified, we focused on creating the ETL pipeline to transform the EHR data for import into the data capture platform. The MUSIC study used the REDCap electronic data capture system to populate and store data across participating hospitals. REDCap provides the user with participant-level manual and bulk data entry via file uploads. Participant-level manual entry is user-friendly and allows researchers to enter data in a form-like format. The files required for bulk data entry must follow a specific encoding to match this format and allow for accurate processing by REDCap.

Our pipeline's results were transformed following these encodings and saved in CSV format as a file ready to upload for bulk data entry in REDCap (Supplemental Table S3). Some examples of the transformations required for our study protocol were: using 1 to codify when a laboratory test result was obtained and 0 when it wasn't, using the value −88 when the EHR measure matched the eCRF expected unit and otherwise noting the units used, and translating non-numeric laboratory test results such as PCR tests (1 for “Detected,” 0 for “Not detected,” and 2 for “Indeterminate.”). These are only some of the transformations required in our study for REDCap to correctly process and display our output.

#### Medications during hospitalization case report form

Observations were filtered to include only medications administered during the patient's MISC hospitalization. Following the study protocol, each medication was encoded following the protocol specifications based on the class and generic name. When required, the administered date was reported.

#### Laboratory values case report form

Observations were filtered to include laboratory values at the five different time points required for the study. MISC hospitalization admission and discharge dates, as well as follow-up visit dates when available, were used to determine what visit a laboratory value corresponded to based on the MUSIC study protocol: first obtained during MISC hospitalization, closest to discharge during MISC hospitalization, two weeks post-discharge, six weeks post-discharge, six months post-discharge.

### Evaluate the performance of the pipelines on real multi-center studies

We applied the pipeline to inpatient EHR data from two different sites and scenarios to demonstrate the feasibility and validity of our approach.

In scenario 1, we aimed to test whether EHR data could successfully be extracted and leveraged to populate eCRFs in site A. In site A, the researchers had not yet manually entered MUSIC study data into REDCap. Thus, we could test whether automated extraction could reduce the burden of data entry for researchers. The evaluation was focused on a subset of patients before the full, automatically extracted dataset was loaded into REDCap. The REDCap development environment was used to perform an exhaustive review of all results in scope for five patients. Specifically, we looked for issues with protocol interpretation, incorrect mapping, and discordant values. Once the clinicians confirmed the concordance of the five random patients, the final data were loaded into the production environment.

In scenario 2, we aimed to measure the concordance of the EHR data with manually entered data and to test the scalability of our pipeline to a completely different site (site B). In site B, the researchers had already entered MUSIC data into REDCap. They had a different clinical data warehouse and EHR model in place. Therefore, we had an opportunity to evaluate our pipeline's applicability in the same study but in a different environment by applying the existing pipeline with minimal modifications to a new set of data. We evaluated the coverage, defined as the percentage of variables in the study found in the EHR across all patients. For site B, we also tested concordance, defined as the percentage of values that were exactly the same between the manual entry and the automatic extraction. The denominator of this percentage was the total number of values entered manually.

Clinicians then manually validated the automatically uploaded data, entered variables not pre-populated automatically, and reclassified variables assigned to a broader category. Although our automated approach required some manual input, the automated data population process significantly decreased the data entry burden and focused data entry effort on those data elements requiring extra attention.

### Role of the funding source

All authors had full access to all the data, accept full responsibility of ensuring accuracy or integrity of any part of the work, approved the final version of the manuscript and agreed to submit it for publication. This study was funded by NIH OTA-21-015E. The funder played no role in study design, data collection, analysis, data interpretation, or this manuscript's writing.

## Results

### Categorization of eCRF variables according to EHR availability and data harmonization

**Fig. 2:**
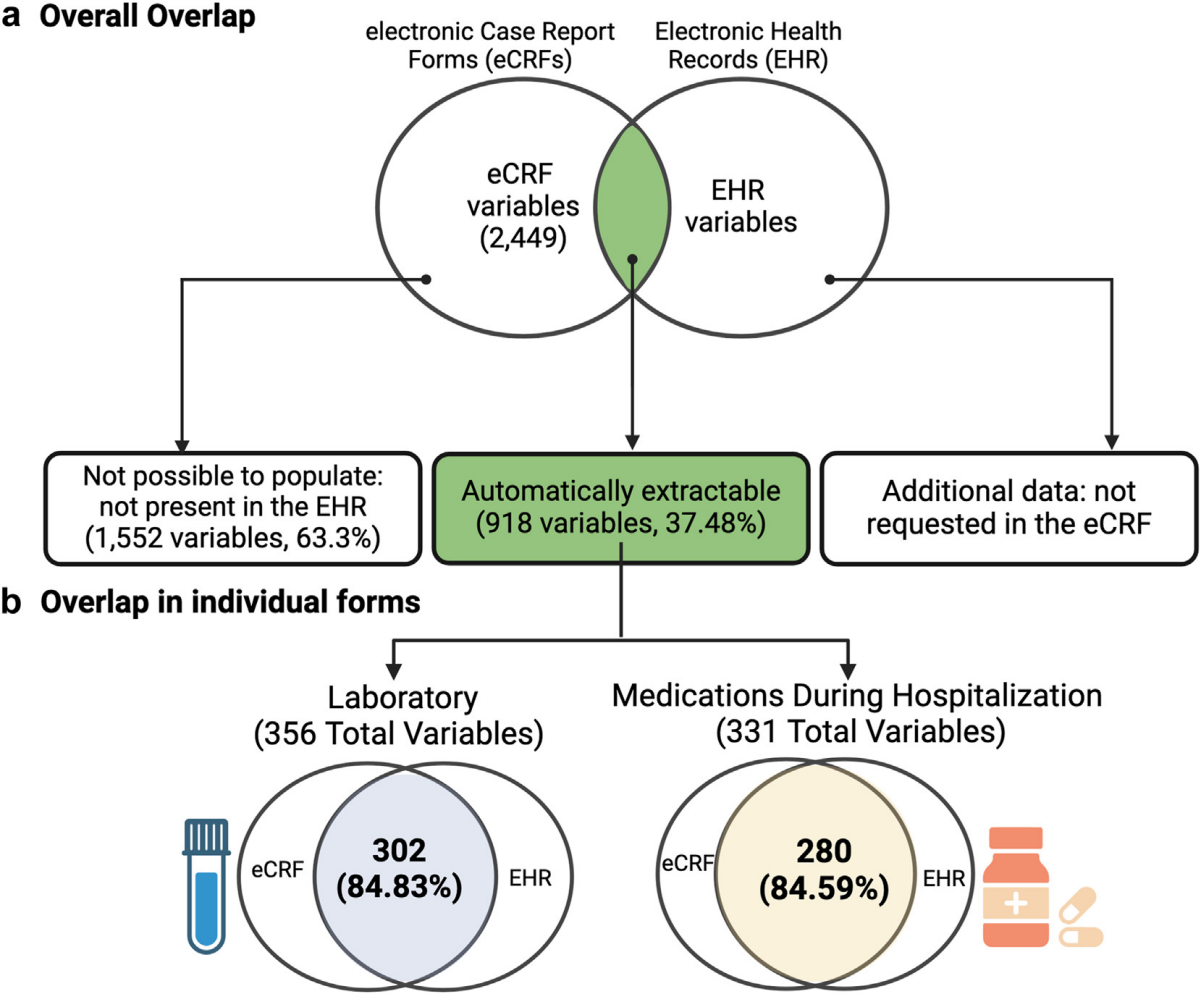
Overlap between the eCRF and EHR variables at the dictionary level. a) Of the 2449 variables in the eCRF, 918 could be automatically extracted from the EHR (area in green); b) The forms with the highest number of variables in the MUSIC study were laboratory (total of 356 variables) and medications during hospitalization forms (total 331 variables). Each Venn diagram represents the percentage of automatically extractable variables from the EHR for each form. Figure created with BioRender.com.

The MUSIC study contained 2449 variables requested by the eCRFs. Of these, 918 (37.48%) were identified as automatically extractable, and 1552 variables (63.37%) as not available in the EHR (Fig. 2a). The laboratory and medications administered during hospitalization eCRFs had the highest prevalence of automatically extractable variables, with 302 (84.83%) of the laboratory variables and 280 (84.59%) of the medications identified as automatically extractable (Fig. 2b). The labs and the medications administered during hospitalization also represented a significant portion–14.54% and 13.52%–of the total variables requested by the MUSIC study (Supplemental Table S4).

The 356 total lab variables arose from 75 laboratory tests, and the 331 medication variables from 28 medication categories (e.g., Antibiotics). Of these, 72 lab tests and at least one medication from all 28 medication categories comprised the identified automatically extractable variables. Examples of values that were not automatically extractable are laboratory test manufacturers and the relationship between a medication and an adverse event. LOINC codes were found for 69 of the 72 laboratory tests. RXNORM codes were found for 282 medications within all 28 medication categories.

### Evaluation of pipeline performance on site A

For site A, our goal was to ensure pipeline results corresponded with manually entered results. This enabled the resulting extracted and transformed data to be loaded into the REDCap production environment. Clinicians reviewed every single result from five random patients, representing 9.09% of the total patients in Site A. Those five patients had approximately 2000 values. After determining 100% concordance, the clinician team felt confident in the complete dataset, and all 29,288 values for all 55 patients in site A were uploaded to REDCap for application in the ongoing MUSIC study. After this confirmation, all 29,288 values were uploaded to REDCap for application in the ongoing MUSIC study.

### Evaluation of pipeline performance on site B

**Fig. 3:**
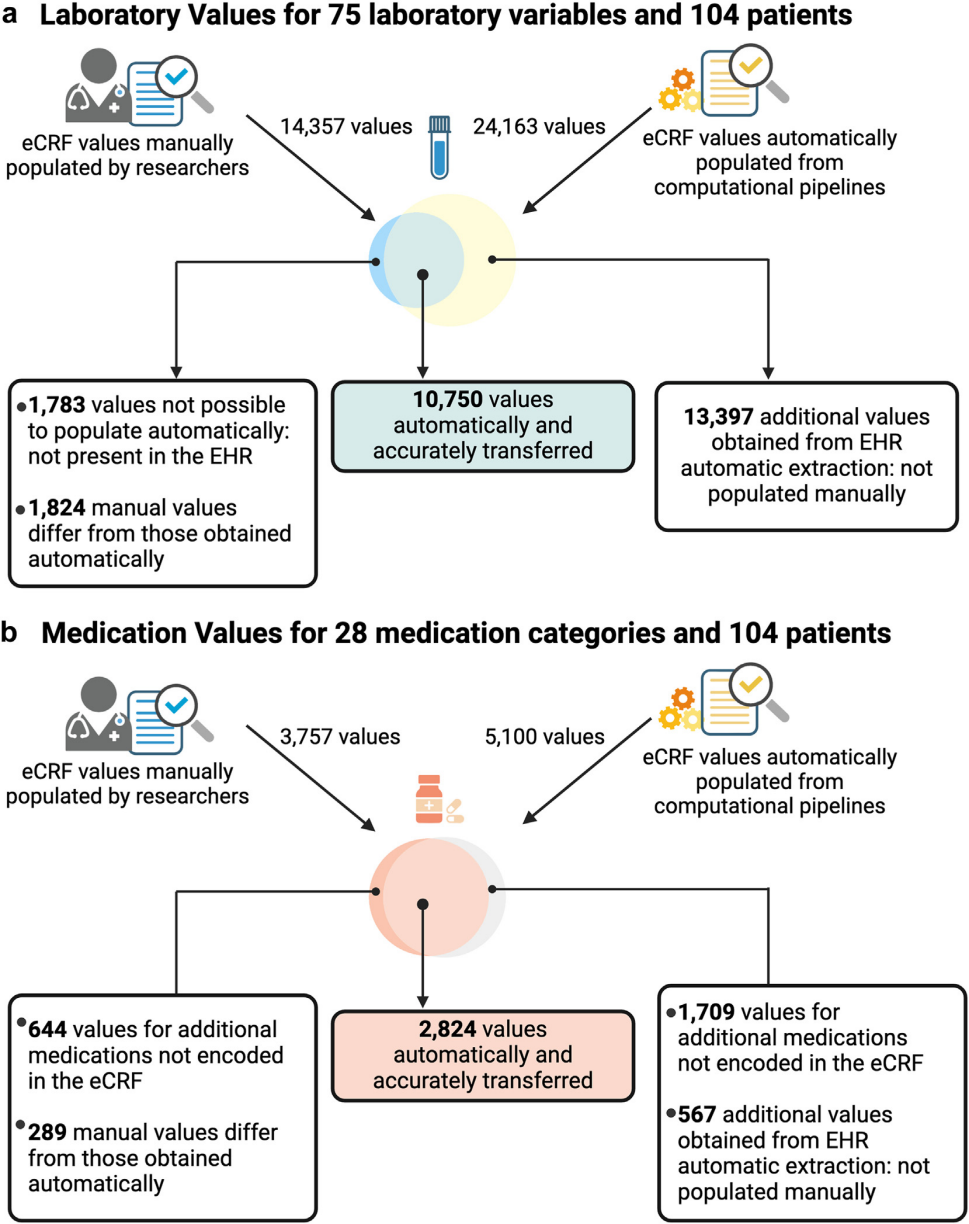
Concordance between manual data entered for site B into REDCap and the automatic data generated after running the pipeline on the same patients. a) Concordance in laboratory eCRF; b) Concordance in medication eCRF. Figure created with BioRender.com.

For site B, our goal was to compare our results with manually entered data. Similarity between pipeline and manual data would represent concordance, albeit not accuracy, since the manually entered values might not have been entirely accurate. We extracted 24,163 laboratory values while researchers at site B manually populated 14,357 laboratory values (Fig. 3a). Out of the 14,357 laboratory values, 12,574 belonged to variables categorized as automatically extractable. Upon comparison 10,750 of our 24,163 automatically extracted laboratory values were identical to the manual data, resulting in a concordance of 85.49% (Fig. 3a). Upon learning that the laboratory form had been filled out at five different time points (Supplemental Figure S3), we measured concordance at each point. Results show that concordance was higher during admission (91.59%) and lower during the follow-up visits (59.98%–40.26%) (Table 1). The remaining manually populated values were either absent in the EHR (1783 values) or differed from the automatically generated ones (1824 values). Automatic extraction also generated an additional 13,397 laboratory values from the EHR that were not manually populated by EHR researchers.

**Table 1:** Concordance (exact matches) of automatically extracted laboratory values compared to laboratory values manually entered by site B researchers, aggregated by visit time point.

Visit time point	Number of manually entered values	Number of automatically extracted values	Concordance
At admission	10,770	13,684	9864 (91.59%)
At discharge	842	10,350	505 (60.98%)
Two-week follow-up	230	169	143 (62.17%)
Six-week follow-up	248	85	85 (34.27%)
Six-month follow-up	380	287	153 (40.26%)

The percentage of concordance is estimated using as denominator the number of manually entered values as the denominator.

We extracted 5100 medication values in site B data, while researchers there manually populated 3757 values (Fig. 3b). Of those, 2824 medications were identical to those extracted automatically, resulting in a concordance of 90.72% (Fig. 3b). Of the remaining manually populated medications, 644 medications were not explicitly requested or encoded in the eCRF but were noted as additional medication by site B researchers (Fig. 3b). The other 289 medications differed from those obtained automatically (Fig. 3b).

## Discussion

In this study, we developed and validated a pipeline for the automatic data population of case report forms, leveraging the robustness and completeness of laboratory and medication records within EHRs. Our method was deployed for two eCRFs across two hospitals participating in an ongoing multi-centric pediatric study. We validated our methodology by comparing our pipeline's automatically extracted, formatted, and loaded results with values manually entered by the research team in one hospital. We further extended our pipeline's application to a second hospital, where instead of manually entering the data, the research team used the output generated by our method as a primary data source for the two case report forms. By streamlining data entry through our computational pipeline, our results mark a significant advancement, moving beyond proof of concept to practical implementation.

Because MUSIC was developed with manual entry in mind, we had first to evaluate which forms would most benefit from automated EHR extraction. To identify the most suitable eCRFs for automated extraction, we weighed the strengths and limitations of EHRs. In agreement with previous studies,^33^^,^^34^ we found that forms with a high volume of laboratory and medication data were more ideal than those focused on clinical characteristics and imaging data, given that the former do not require clinical interpretation and exhibit as structured data in the EHR. Forms collecting variables that were not accurately translated into structured EHR codes (e.g., if a medication was related to an adverse event), required interpretation (e.g., the electrocardiogram-related forms) or were unstructured text (e.g., open-ended questions collecting other medications) required an extensive manual review process from clinicians and were discarded in this study. These and other types of variables, including administrative information or the manufacturers of specific laboratory tests, cannot benefit from the automatic pipeline presented in this study since the data are either not stored in the EHR data warehouse or require additional interpretation. However, future implementation of techniques such as Natural Language Processing, could expand this pipeline and complement the structured EHR data with information from clinical notes and discharge summaries.^35^

Additionally, we considered the visit type (e.g., during hospital admission, follow-up, outpatient) required by the eCRF. EHR completeness and accuracy during hospital admissions are usually higher than outpatient visits, and different visit types may have been recorded differently in the EHR. For example, it was not possible for us to accurately populate the eCRF with medications prescribed before and after hospital admission, even though those variables were highly prevalent in the EHR. This is because prescribed medications were primarily recorded as clinical notes rather than structured data fields. No one could guarantee that a patient took the noted medication.

This study focused on two structured data types (laboratory values and medications). The laboratory and medications during admission eCRFs represented 28% of the total variables collected in the MUSIC study and were among the most time-consuming forms. Although the labs and medication concepts were structured and codified, we faced a challenge: the lack of standardized vocabularies in the data warehouses. The quality of the pipeline outputs relied heavily on the quality of the mappings, which allow data integration and interoperability but pose some challenges. Data mapping consists of finding the most accurate mapping for each variable in the most suitable ontology. However, when going through the mapping process, common scenarios include missingness, since not all the variables can always be mapped^36^ and one-to-many mappings, when multiple terms in an ontology could fit for the same variable. Given the one-to-many mappings found between dictionaries, to guarantee the accuracy of our mappings, we generated a summary of the variables derived from the eCRFs and patient-level data, comparing observations gathered at each institution to expectations of the eCRF (Supplemental Table S1). Such a comparison allowed us to ensure that all mappings were correct and current, providing confidence in the quality of the final eCRF-EHR mapping. The clinicians in contact with the enrolled patients checked general trends, comparing the total number of patients and observations found for a certain lab with their knowledge of how commonly those panels were requested. Labs commonly requested for MIS-C patients were found to have a high prevalence for all patients; when no EHR data were found for a lab, clinicians confirmed that the lab did not apply to any MUSIC patients. The observed date range allowed us to identify the most up-to-date EHR data. We discarded any mappings that were present in the EHR, but not relevant during the MUSIC study period. We compared the range of each lab value to identify outliers and checked the recorded units to ensure they were as expected. In this way, the summary table provided insight into broader data issues and helped us identify common data harmonization challenges The challenges encountered during these quality control steps encompass unit mismatch, outlying values, missing data, scattered data sources for the laboratory tests, typos, misclassifications, and route-dependent data for the medications. To overcome these limitations, we implemented unit standardization, outlier detection, clinician consultation, prioritization of recent data, and alignment between medication routes in the EHR and eCRF.

To evaluate whether the pipeline was robust and scalable to all participants, the clinicians at site A manually checked a random sample of patients. For site B, we used the manual data entered for comparison. We found that the concordance of the results for the laboratory test form was highly influenced by the time points when the data had to be collected. For admission and discharge, the source of discrepancy tended to be the multiplicity of values available for the same time point (e.g., albumin collected at three different times during the admission date). Only one value per time point per patient could be reported in the MUSIC study eCRF. In the manual approach, the clinicians entering the data selected a random value if no significant difference was observed. This approach was not feasible when writing the automatic pipeline, as clear guidelines and rules were required. Therefore, in cases with multiple values, we selected the first value recorded. In the cases we manually reviewed, there were no clinical differences in choosing one value above another. However, this difference in approach for manual versus automatic entry still led to discrepancies in concordance when the values did not match precisely. For the follow-up visits, we could not ascertain the actual dates. Based on the clinical expertise, we used a window of time, adding flexibility. Another source of discrepancy was the lack of consistency in location of data collection. For example, the pipeline ran on inpatient data. Some follow-up visit information was recorded in the EHR as an outpatient visit, however. This bias towards data recorded as an outpatient visit is visible in Table 1, as the concordance of our pipeline dramatically decreased following patient hospitalization. It is possible that this was due to the approximations taken to select values. For medication values, we found differences, for example, in the reporting of class versus generic name. These discrepancies do not indicate that either value needs to be corrected. The feedback from the teams of researchers allowed for a more efficient data extraction process, quickly solving issues and discrepancies in the automated extraction results.

Both manual data collection and automatic extraction have potential and known inaccuracies. Manual data collection is error-prone, while automatic extraction may not identify the most clinically meaningful results. As a proxy to measure the quality of the pipeline, we measured the concordance by comparing the manually entered versus the automatically extracted data. Nevertheless, it should be noted that manually entered data can also be inaccurate or contain errors, even following clinician review. The goal of this work was not to generate the “gold standard” of patient data, but to prove how effectively an automatic pipeline can support the data entry process.

The overall premise of this study stems from the increased use of EHR as a valuable resource for epidemiological and observational research. As an incentive, regulatory agencies such as the Food and Drug Administration (FDA),^1^^,^^37^ medical organizations, societies, and foundations are acknowledging the potential of EHRs in biomedical research and encouraging EHR integration with electronic data capture systems.^38^^,^^39^ The impact of leveraging EHR data for research extends to researchers, patients, and regulatory bodies, addressing a critical gap in current practices. Primarily, it directly benefits researchers and study coordinators by reducing the time and resources required for data entry while increasing potential scope and impact. Additionally, clinical study participants benefit from improved data quality and consistency, leading to quicker solutions and better health outcomes.

Currently, the variability of EHR systems, data models, and data standards are a barrier when implementing automatic data extraction. The adoption of universal common data models, such as ODHSI OMOP,^23^ i2b2^40^ or PIC-SURE^41^ could speed up the design and implementation of automatic data approaches like the one described in this study. While additional research is required to continue exploring and refining the methods presented here, nonetheless, EHRs offer a rich source of routinely collected patient-level data that can be leveraged to automatically provide high-quality information for clinical trials. Developing eCRFs considering automatic data extraction can advance research in future studies, and considering the strengths and limitations of the EHR will be a valuable advancement in eCRF development. Studies will benefit maximally from automatic approaches like the one described in this study by using structured data rather than free text when possible and standardized ontologies and common data models when available. Developing eCRFs considering automatic data extraction can advance research in future studies by improving data extraction and integration methods while ensuring the utility of structured clinical data. The only requirement is having an existing clinical data warehouse in place. By automating data entry for some of the most time-consuming variables, we present a transformative solution to a critical issue, enhancing research efficiency and cost-effectiveness. Our open-source approach transcends platform-specific limitations, ensuring broad applicability and generalizability.

## Contributors

AGS, SM, AD, CW, and PA contributed to the design and conceptualization of the study.

AGS, SM, AD, SM, KJD, FS, PP, AD, JCL, DMP, LSS, DT, JWN and PA contributed to data collection. AGS, SM, AD, PP, SM, AD, JMZ, and PA contributed to data analysis or interpretation. All authors contributed to drafting or revising the work critically for important intellectual content and approved the final version. All authors are responsible for all aspects of the work in ensuring that questions related to the accuracy or integrity of any part are appropriately investigated and resolved.

## Data sharing statement

This study determined requirements and evaluated a pipeline based on EHR records from Boston Children's Hospital and Texas Children's Hospital. These records are confidential patient information that cannot be shared. The underlying code for this study is available on GitHub and can be accessed via this link https://github.com/hms-dbmi/music-ecrf-harmonization under an open-source Apache 2.0 license.

## Declaration of interests

The authors declare no competing interest.
