# Supplementary material for: Development and validation of an open-source pipeline for automatic population of case report forms from electronic health records: a pediatric multi-center prospective study
Source: eBioMedicine. 2024 Sep 16;108:105337. doi: 10.1016/j.ebiom.2024.105337 (PMC11421260; doi:10.1016/j.ebiom.2024.105337)
Supplement: Supplementary Tables and Figures [file mmc1.docx]

**Supplementary Tables and Figures**

**Supplemental Table 1: Sample quality control summary of the variables extracted, using three laboratory tests.**

**Supplemental Table 2: Summary of automatically extracted values for labs and medications during hospitalization.**

**Supplemental Table 3: Example of REDCap input format for the laboratory eCRF.**

**Supplemental Table 4: eCRFS from MUSIC study: an overview of the number of variables per form.**

**Supplemental Figure 1: Data extract: files and format descriptions.**

**Supplemental Figure 2: Overview of the dictionary mapping.**

**Supplemental Figure 3: Overview of the information collected for each laboratory test and the different time points in which this information was collected for each patient.**

# **Supplemental Table 1: Sample quality control summary of the variables extracted, using three laboratory tests.** This table summarizes the information obtained for each variable from the eCRF (columns 1 to 6, in gray); the summary of the variables based on the information obtained from the EHR (columns 7- 14, in yellow); the checks and potential questions from the EHR experts (columns 15 and 16, in green) and the checks and potential comments from the clinicians (columns 17 and 18, in purple)

| **eCRF and dictionary** | | | | | | **Summary based on site EHR data** | | | | | | | | **To be completed by the EHR and Data Transformation Experts** | | **To be completed by the Clinical Research Experts** | |
| --- | --- | --- | --- | --- | --- | --- | --- | --- | --- | --- | --- | --- | --- | --- | --- | --- | --- |
| Form Name | Section Header | Variable | Min.  Exp.  Value | Max.  Exp.  Value | Units | concept description | site concept code | Observed date range | Number Of Patients | Number  of Observ. | Observed Minimal Value | Observed Maximum Value | Observed Units | Units Check | Question | Check | Comments |
| Laboratory | Creatinine (mg/dL) | creatine value | 0.12 | 12 | mg/dL | Cre | LB:48595 | 12-Dec-09 / 5-April-21 | 43 | 562 | 0.1 | 3.15 | mg/dL | OK |  |  |  |
| Laboratory | Albumin (g/dL) | albumin value | 0.5 | 15 | g/dL | Albumin | LB:36258 | 15-Dec-08 / 30-Jan-21 | 51 | 419 | 1.9 | 5.1 | g/dL | OK |  |  |  |
| Laboratory | APTT or PTT (seconds) | aptt/ptt value | 1 | 100 | seconds | PTT | LB:58469 | 22-Apr-09 / 4-Apr-21 | 39 | 238 | 20 | 153.8 | sec | OK | Is this a correct mapping? | Yes |  |

# **Supplemental Table 2: Summary of automatically extracted values for labs and medications during hospitalization.**

| **Electronic Case Report Form** | **Number of values extracted from site A (55 patients)** | **Number of values extracted from site B (104 patients)** |
| --- | --- | --- |
| Laboratory Values  (from 75 laboratory tests) | 27,684 | 24,163 |
| Medications During Hospitalization  (from 28 medication categories) | 1,604 | 3,309 |

#

# **Supplemental Table 3: Example of REDCap input format for the laboratory eCRF.** The first column represents the patient identified. Columns 2-5 are related to the eCRF & registry platform. Columns 6-9 exemplify how the information is saved for one specific laboratory value, Sars CoV2 nt-PCR, with a separate value for the visit type, whether the laboratory test was obtained, the date, and the laboratory test result. Values are defined by the study data dictionary.

| **record_id** | **redcap_event_name** | **redcap_repeat_instrument** | **redcap repeat instance** | **redcap_data_access_group** | **lab_values_visit** | **sars_cov2ntpcr_obtained** | **sars_cov2ntpcr_date** | **sars_cov2ntpcr_value** |
| --- | --- | --- | --- | --- | --- | --- | --- | --- |
| 160001 | repeating_forms_arm_1 | laboratory_values | 1 | 160_medical_univer | 1 |  |  |  |
| 160001 | repeating_forms_arm_1 | laboratory_values | 2 | 160_medical_univer | 1 | 1 | 6/2/20 | 1 |
| 160001 | repeating_forms_arm_1 | laboratory_values | 3 | 160_medical_univer | 1 | 1 | 8/16/20 | 1 |
| 160001 | repeating_forms_arm_1 | laboratory_values | 4 | 160_medical_univer | 2 | 0 |  |  |
| 160001 | repeating_forms_arm_1 | laboratory_values | 5 | 160_medical_univer | 23 | 0 |  |  |

**Supplemental Table 4: eCRFS from MUSIC study: an overview of the number of variables per form.**

| **Electronic Case Report Form Name** | **Automatically extractable variables (% from the variables in the form)** | **Variables per form**  **(% from the total variables in the study)** |
| --- | --- | --- |
| laboratory_values | 302 (84.83%) | **356(14.54%)** |
| additional_medications_during_hospitalization | 280 (84.59%) | **331(13.52%)** |
| medications_before_and_after_hosp | 29 (80.56%) | 36(1.47%) |
| clinical_presentation_and_status_on_admission | 69 (69%) | 100(4.08%) |
| heart_transplant | 2 (66.67%) | 3(0.12%) |
| demographics | 20 (55.56%) | 36(1.47%) |
| chest_x_ray | 7 (43.75%) | 16(0.65%) |
| cardiac_mri | 4 (40%) | 10(0.41%) |
| chest_ct | 8 (40%) | 20(0.82%) |
| cardiac_clinical_information | 20 (33.9%) | 59(2.41%) |
| hospital_and_clinical_course | 64 (32.16%) | 199(8.13%) |
| death | 3 (30%) | 10(0.41%) |
| summary_evaluation_of_kawasaki_disease_features | 5 (29.41%) | 17(0.69%) |
| non_cardiac_organ_systems_review | 27 (28.72%) | 94(3.84%) |
| electrocardiogram | 8 (26.67%) | 30(1.22%) |
| exercise_test | 10 (25%) | 40(1.63%) |
| screening | 3 (25%) | 12(0.49%) |
| annual_cardiac_clinical_information | 11 (23.4%) | 47(1.92%) |
| doses_of_steroids_aspirin_lmwh_during_hosp | 23 (21.5%) | 107(4.37%) |
| cardiac_ct | 4 (13.79%) | 29(1.18%) |
| end_of_study | 1 (11.11%) | 9(0.37%) |
| bacterial_and_fungal_cultures | 4 (9.09%) | 44(1.8%) |
| local_echocardiogram | 6 (7.79%) | 77(3.14%) |
| sarscov2_and_respiratory_panel_testing | 3 (5.17%) | 58(2.37%) |
| baseline_health_status | 5 (4.42%) | 113(4.61%) |
| additional_findings_and_events | 0 (0%) | 53(2.16%) |
| additional_informed_consent | 0 (0%) | 9(0.37%) |
| adverse_event_follow_up_report | 0 (0%) | 42(1.71%) |
| adverse_event_initial_report | 0 (0%) | 41(1.67%) |
| ambulatory_monitoring | 0 (0%) | 27(1.1%) |
| annual_follow_up_info_and_vital_status | 0 (0%) | 23(0.94%) |
| annual_general_health | 0 (0%) | 13(0.53%) |
| eligibility | 0 (0%) | 10(0.41%) |
| entry_into_healthcare_system | 0 (0%) | 5(0.2%) |
| follow_up_information_and_vital_status | 0 (0%) | 15(0.61%) |
| functional_status_score | 0 (0%) | 9(0.37%) |
| general_health | 0 (0%) | 12(0.49%) |
| hospital_readmission | 0 (0%) | 88(3.59%) |
| informed_consent_and_enrollment | 0 (0%) | 13(0.53%) |
| medical_monitor_review | 0 (0%) | 22(0.9%) |
| new_family_history | 0 (0%) | 118(4.82%) |
| parent_1_biospecimen_sample | 0 (0%) | 8(0.33%) |
| parent_2_biospecimen_sample | 0 (0%) | 8(0.33%) |
| participant_biospecimen_sample | 0 (0%) | 8(0.33%) |
| promis_global_health | 0 (0%) | 33(1.35%) |
| protocol_deviation | 0 (0%) | 8(0.33%) |
| status_and_disposition_at_discharge | 0 (0%) | 31(1.27%) |


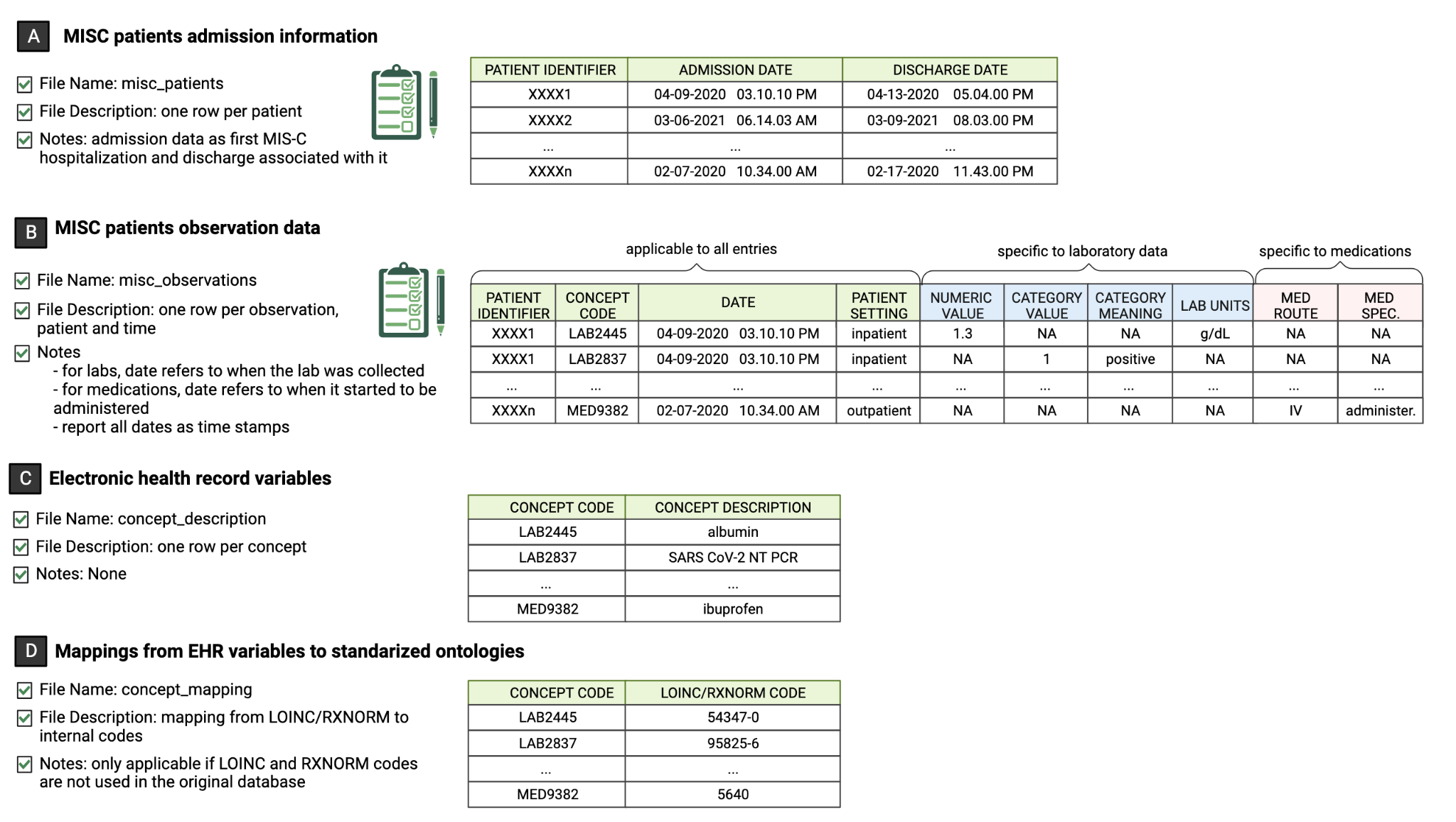


# **Supplemental Figure 1: Data extract: files and format descriptions.** Information shared with each of the participating sites.

#
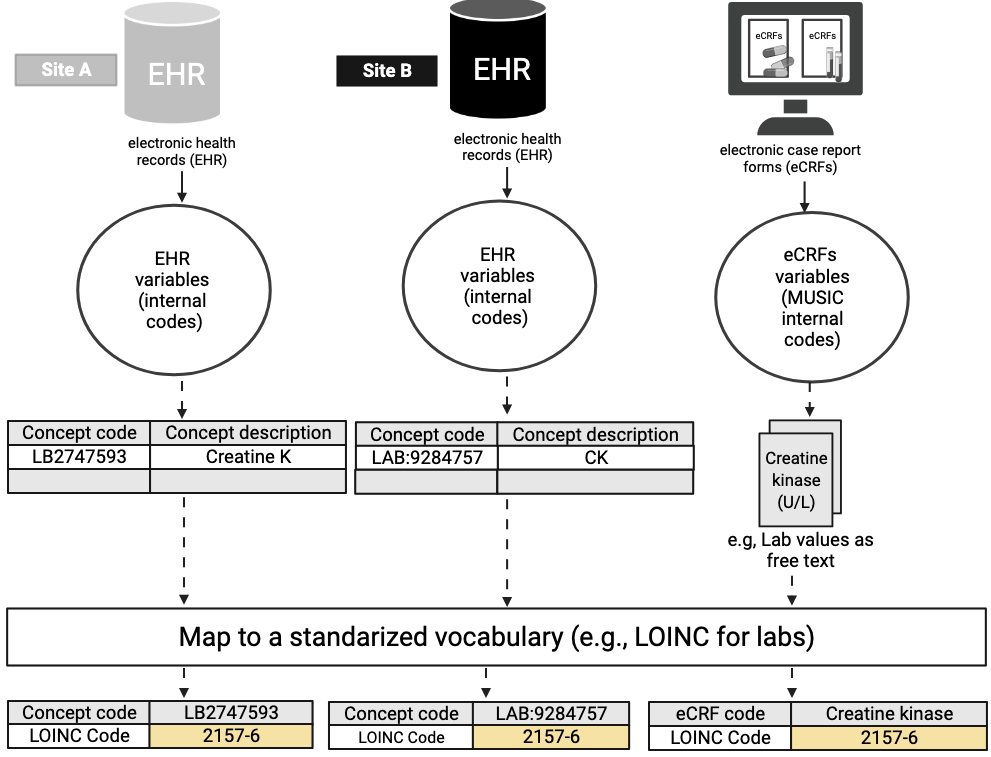
**Supplemental Figure 2: Overview of the dictionary mapping.** Example of vocabulary mapping between the eCRF dictionary and two potential EHR dictionaries for creatinine kinase. Figure created with BioRender.com.


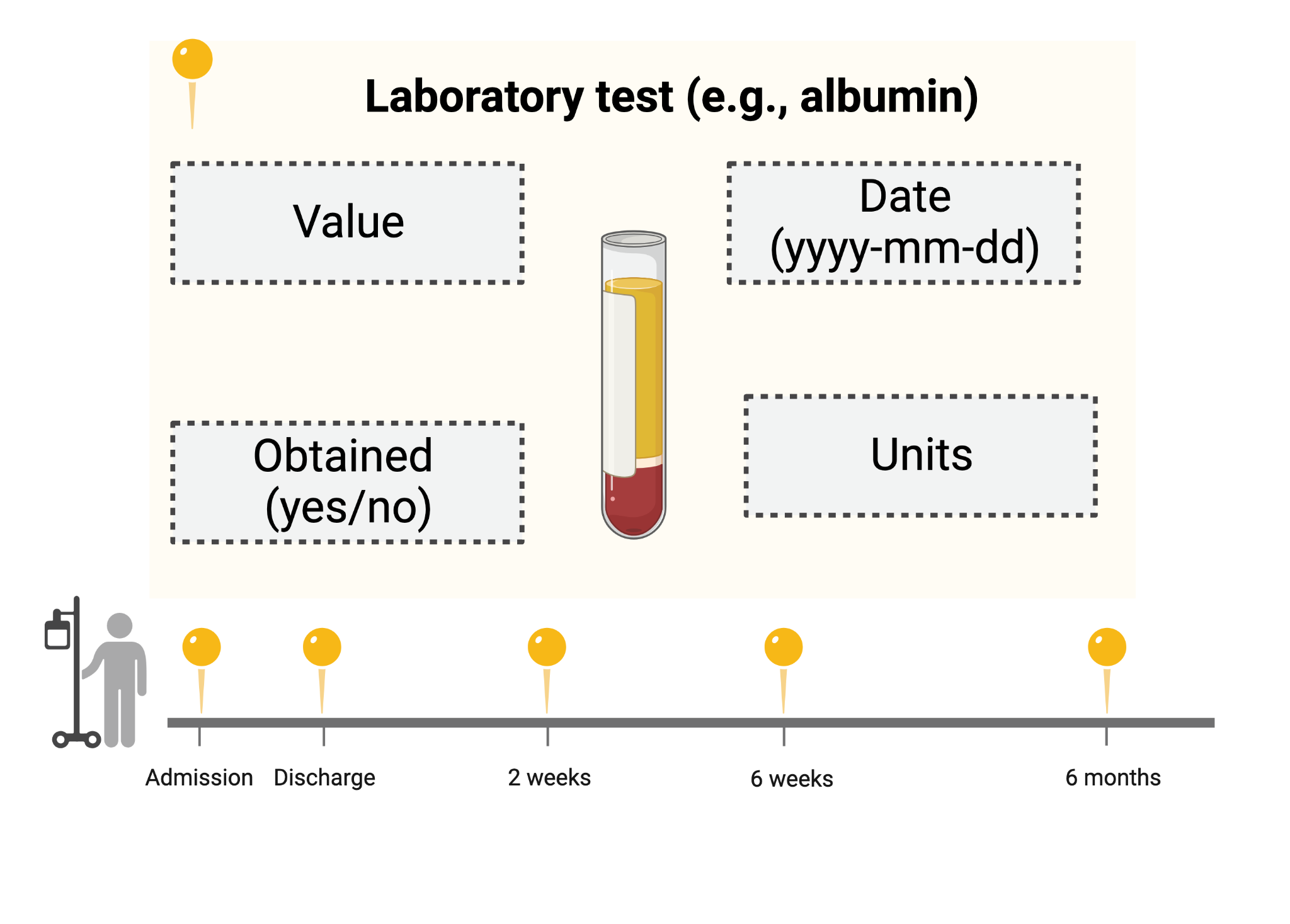


# **Supplemental Figure 3: Overview of the information collected for each laboratory test and the different time points in which this information was collected for each patient.** Figure created with BioRender.com.
